# Neurotoxic Shellfish Poisoning

**DOI:** 10.3390/md20080021

**Published:** 2008-07-12

**Authors:** Sharon M. Watkins, Andrew Reich, Lora E. Fleming, Roberta Hammond

**Affiliations:** 1 Aquatic Toxins Program, Division of Environmental Health, Florida Department of Health, Tallahassee, FL 32399-1712, USA; 2 NSF NIEHS Oceans and Human Health Center, Rosenstiel School of Marine and Atmospheric Sciences, University of Miami, FL 33136, USA; 3 Food and Waterborne Disease Program, Division of Environmental Health, Florida Department of Health, Tallahassee, FL 32399-1712, USA

**Keywords:** Neurotoxic Shellfish Poisoning, NSP, brevetoxins, harmful algal blooms (HABs), *Karenia brevis*, epidemiology, human health, red tide

## Abstract

Neurotoxic shellfish poisoning (NSP) is caused by consumption of molluscan shellfish contaminated with brevetoxins primarily produced by the dinoflagellate, *Karenia brevis.* Blooms of *K. brevis*, called Florida red tide, occur frequently along the Gulf of Mexico. Many shellfish beds in the US (and other nations) are routinely monitored for presence of *K. brevis* and other brevetoxin-producing organisms. As a result, few NSP cases are reported annually from the US. However, infrequent larger outbreaks do occur. Cases are usually associated with recreationally-harvested shellfish collected during or post red tide blooms. Brevetoxins are neurotoxins which activate voltage-sensitive sodium channels causing sodium influx and nerve membrane depolarization. No fatalities have been reported, but hospitalizations occur. NSP involves a cluster of gastrointestinal and neurological symptoms: nausea and vomiting, paresthesias of the mouth, lips and tongue as well as distal paresthesias, ataxia, slurred speech and dizziness. Neurological symptoms can progress to partial paralysis; respiratory distress has been recorded. Recent research has implicated new species of harmful algal bloom organisms which produce brevetoxins, identified additional marine species which accumulate brevetoxins, and has provided additional information on the toxicity and analysis of brevetoxins. A review of the known epidemiology and recommendations for improved NSP prevention are presented.

## 1. Introduction

Neurotoxic Shellfish Poisoning (NSP) is a disease caused by the consumption of molluscan shellfish contaminated with brevetoxins; these are a group of more than ten natural neurotoxins produced by the marine dinoflagellate, *Karenia brevis* (formerly known as *Gymnodinium breve* and *Ptychodiscus brevis*) [1]. Brevetoxins comprise a group of toxins with primarily neurological and gastrointestinal effects. Cases of NSP are relatively uncommon and this paper provides a review of the known epidemiology and recent findings. *K. brevis* is naturally occurring in the Gulf of Mexico, Caribbean Sea and along New Zealand coasts; it regularly produces blooms along the coasts of Florida and Texas. This environmental phenomenon is a harmful algal bloom (HAB) known as “Florida red tide” [2, 3]. Blooms of *K. brevis* can cause the ocean to appear red, brown, or simply darkened due to the dense aggregation of cells which often includes several species of unicellular algae. Blooms are usually accompanied by massive fish kills and mortalities in marine mammals and sea birds [4–8]. These die-offs can be the first indicators of a red tide bloom event. *K. brevis* is only one of the marine species of dinoflagellates that produces brevetoxins (e.g., *K. mikimotoi, K. brevisulcata, K. selliformis, and K. papilionacea*), but it remains the best studied to date. Recent evidence for brevetoxins production by raphidophytes (e.g., *Chatonella spp*.) has been documented as well [9].

Red tide is a generic colloquial term for a number of different types of microscopic algal blooms occurring throughout the world; the more scientifically accepted term is “harmful algal bloom” or “HAB.” Blooms of *K. brevis* were first reported in 1844 in Florida predating the rapid economic growth and development of the mid to late twentieth century by many decades [10]. Blooms were once considered to be sporadic and seasonal, but historical records demonstrate that these blooms have occurred in Florida almost annually in the years since the 1940s [11]. Although more frequent in late summer and early fall, Florida red tide has been documented to occur in almost every month of the year [12]. Blooms may disperse in a matter of weeks or may be present for many months at a time; in 2006, a bloom off the coast of Sarasota (Florida) lasted over 12 months.

Much of current research is focused on understanding the relationship between nutrients and their possible role in bloom initiation, growth and sustenance [13–16]. Other areas of interest include the possible transport and discharge of dinoflagellate cysts in the ballast water of ships; the possible influence of changes in currents, weather patterns and ocean temperatures from climate change; and the possible atmospheric transport and deposition of iron rich Saharan dust [17–25]. On a global scale HABs, including *K. brevis*, may be increasing in frequency, duration and geographic range in all aquatic environments [26–28].

## 2. Geographical Areas

Blooms of *Karenia brevis* are considered endemic to the Gulf of Mexico, particularly off the southwestern coast of Florida. The first recorded blooms from this area were in the 1840s, although earlier Spanish records allude to red tide-like conditions and events during the mid 1500s describing fish die offs, “bad air’ and discolored water [10, 29]. These early records may be an actual description of a red tide event, or may be an account of fish die-off related to low oxygen concentration in shallow waters. Red tides also affect the Florida panhandle, the Atlantic coast of Florida, Texas, and all the coastal areas bordering the Gulf in Mexico. Ocean currents have transported *K. brevis* blooms up the Atlantic coast. The largest reported outbreak of NSP in the US occurred in North Carolina after *K. brevis* was carried into that region [17, 30, 31].

There are numerous other *Karenia spp*. found in the Gulf of Mexico and around the world (particularly New Zealand) regularly associated with blooms. Most produce brevetoxins, gymnodimine, karlotoxins, and other potent natural toxins [32–35]. The largest recorded outbreak of NSP occurred in New Zealand in 1992 – 1993 due to consumption of cockles and green shell mussels; oysters have also been implicated in the New Zealand outbreak. Review of this outbreak implicated *K. mikimotoi* as the likely causative agent, but other suspect species were also present in the bloom [33, 36–39].

In 2000, a bloom of *Chattonella cf. verruculosa* found in Delaware bays and creeks was accompanied by massive fish kills (menhaden). This bloom contained elevated levels of brevetoxins, although neither *K. brevis* nor *K. brevis*-like species were found. *Chattonella* had previously been associated with fish kills in other northern waters, but never before in US waters. This particular organism was found to be nearly as toxic as *K. brevis* and more toxic than the previously described *Chattonella* species [9]. A full description of this new species and its toxin-producing abilities is underway [40]. Descriptions of other fish-killing species which produce toxins (similar to brevetoxins) have been reported from Japan and Australia in recent years (although with no associated reports of NSP) [26]. These findings further increase the potential areas of the world at risk for NSP as *K. brevis* is found in new regions, and the other harmful algal bloom species, such as *Chattonella cf. verruculosa*, are found to produce brevetoxins. Current testing guidelines and practices for molluscan shellfish often rely on collection and toxin analysis of the dominant commercial species in the shellfish test zone. This type of sentinel warning may not be sufficient for the protection of public health as brevetoxins are found to be produced by other species in different geographic areas.

## 3. Sources of Human Exposure to Brevetoxins

Shellfish (such as clams, oysters, whelks, mussels, conch, coquinas, and other filter-feeding molluscs) accumulate biotoxins according to their natural food chain patterns. Brevetoxins have been reported in many shellfish species, but the most common sources of human exposure are through the ingestion of commonly harvested clams, oysters and mussels. In general, shellfish themselves do not appear to be affected by the accumulation of brevetoxins. Depuration time of brevetoxins in shellfish varies, but is typically within two to eight weeks, although reports of much longer retention (nearly one year post bloom) have been documented [41–44].

Planktivorous finfish have recently been shown to retain brevetoxins in the muscle, gut and organs, but at much lower concentrations than in molluscs [5, 20, 45–47]. Public health implications of this finding are not able to be evaluated as additional research on the human toxicity of these lower doses is needed. Current guidelines do not limit the consumption of fillets from healthy finfish harvested during or after a red tide. The practice of eating whole fish (in some Asian communities and other cultures) may put individuals at risk for brevetoxin-poisoning as the highest levels of brevetoxins in finfish have been found in the fish liver and stomach contents [47,48]. Brevetoxins may persist in finfish, particularly livers, for more than a year after bloom cessation [47].

Like many marine toxins, brevetoxins are not diminished by rinsing, cleaning, cooking, or freezing, and the toxins cannot be detected by taste or smell. The inability to easily detect toxins and the presence of brevetoxins in these human food sources (shellfish and finfish) past bloom cessation potentially puts consumers of non-commercial shellfish (and consumers of certain whole finfish) at an increased risk for NSP.

## 4. Biological Mechanism of Brevetoxins

The toxin group responsible for NSP, brevetoxins, is comprised of more than 10 lipid soluble cyclic polyethers with a molecular weight of approximately 900 [49, 50]. A number of analogs and metabolites have also been identified. The polyethers are identified using a notation system PbTx 1-*n* based on the numbering system proposed by Shimizu [51]. All derivatives of natural toxins explored to date are less toxic than the parent compounds of PbTx-1 and PbTx-2 [52]. There are two structural backbones for brevetoxin molecules (termed “backbone A” or “backbone B”). These backbones are characterized as relatively linear with a bend mid-molecule exhibiting lactone functionality in the A ring with a series of rings that then form the ladder-like structure. Both polyether backbones terminate in a reactive, α,β unsaturated aldehyde side chain [52, 53].

Brevetoxins bind with high affinity to receptor site 5 on the voltage-gated sodium channel (VGSC), and induce a channel mediated Na+ ion influx [54, 55]. Neuro-excitation results from the nerve membrane depolarization and spontaneous firing. In some cases only the nerve is depolarized, but both nerve and muscle depolarization have been noted [49, 56–61].

Given their lipid solubility, brevetoxins pass through cell membranes including the blood-brain barrier. Brevetoxins are rapidly absorbed and distributed throughout the body, and are metabolized in the liver [62,63,64]. They are primarily removed in the bile within the first 48 hours, but urinary excretion plays a role after this time as well. Chronic and sub chronic toxicity of brevetoxins are unknown, as mammalian studies have explored acute effects. Radio-labeling showed distribution to skeletal muscle, liver and intestine with little activity elsewhere [52, 62, 63]. Sublethal doses administered orally in rats exhibited wide distribution to all organs, highest concentrations were found in the liver up to 8 days after exposure [64]. The LD_50_ (of PbTx-3) in mice ranges from 0.170 mg/kg body weight intraperitoneally, 0.094 mg/kg body weight intravenously, and 0.520 mg/kg body weight orally [52, 65–67]. Metabolites have been found in human urine samples collected within hours post ingestion, but are absent in samples 3–4 days post ingestion [68, 69]. Brevetoxins appear to have a short half-life in serum, but the total body clearance is estimated to take days [62, 63, 70]. Chemical structure, biological mechanisms and other toxicological studies are described in more depth in recent research and review papers [52, 62, 63, 70–81].

Historically, NSP toxins in shellfish had been assumed to be brevetoxins unaltered during accumulation in the molluscs. It has now been shown that molluscan metabolites of parent brevetoxins contribute as the causative agents for NSP [33, 37–39, 42, 50, 68, 82–85]. In the future, these molluscan metabolites may need to be accounted for in alternate methods being considered for regulatory testing and in the monitoring of shellfish beds to more accurately describe the true toxicity of the shellfish.

A recent evaluation of human toxicity to establish an acute reference dose (acute RfD) (Joint FAO/WHO/IOC ad hoc Expert Consultation on Biotoxins in Molluscan Bivalves) concluded there was insufficient human toxicity data to complete a risk assessment, and that further research on brevetoxins and their metabolites (particularly their oral potencies) needs to be performed before an acute RfD could be established [70].

## 5. Diagnosis and Treatment

The diagnosis of NSP is based upon the clinical presentation, plus a carefully elicited history that includes recent consumption of molluscan shellfish. Symptoms begin within a few minutes up to 18 hours after the consumption of contaminated shellfish. A mean time to onset of 3–4 hours has been reported in the few documented outbreaks [30, 68, 69, 86, 87].

NSP causes a range of signs and symptoms, both neurological and gastrointestinal. Most individuals report multiple symptoms. Mild to moderate nausea, vomiting and diarrhea are often reported, although these may not be the chief presenting complaints. Victims of NSP most frequently describe numbness and tingling in the lips, mouth and face, as well as numbness and tingling in the extremities. These paresthesias may be minor to fairly severe, and have been described as feeling like the “nerves are on fire or ants are crawling and biting all over.” Ataxia, overall loss of coordination, and partial limb paralysis may also occur. The reversal of hot and cold sensation has been reported as well, a symptom shared with ciguatera poisoning [88]. Slurred speech, headache, pupil dilation, and overall fatigue are also commonly reported. Victims have been described as appearing disoriented and possibly intoxicated. Throat tightness and chest heaviness have also been reported. A few individuals have had respiratory discomfort and distress, with a handful of cases requiring ventilatory support. The characteristic cluster of both gastrointestinal and neurological symptoms occurs at approximately the same time, with the neurological symptoms lasting longer than the gastrointestinal discomfort [58–61, 67, 68, 89–92].

NSP has been described as similar to a mild case of Paralytic Shellfish Poisoning (PSP). PSP produces similar symptoms, but with a rapid and more severe clinical progression that often involves paralysis, respiratory distress and death if undiagnosed and untreated. Classifying NSP as mild may be misleading as NSP symptoms can also become quite serious (although there are no documented deaths due to NSP). Despite dramatic presentation, victims reportedly recover within 2–3 days, and most often within the first 48 hours. There are no long-term or chronic effects of NSP documented in the literature, although there has been no long term follow-up of victims, and one anecdotal case report includes lingering symptoms [87]. Victims of ciguatera, another disease caused by marine toxin poisoning by ciguatoxin, frequently have symptoms that can linger from weeks to months. Longer sequela could be possible as brevetoxins and ciguatoxins are structurally very similar and have similar modes of action [55, 88]. General descriptions of NSP may be found in the following references [31, 88, 93–99].

Treatment for NSP involves mainly supportive care. Fluid replacement, observation of respiratory functions, and the administration of sedatives and pain mitigation are the main tasks as there is no current specific antidote available for brevetoxins. Gastrointestinal decontamination with activated charcoal for patients presenting within the first 4 hours post ingestion has been recommended [67, 95, 98–100], but there is no clinical evidence supporting a significantly better or lessened course of illness. Given the similar toxin structure between brevetoxin and ciguatoxin, mannitol (the recommended treatment in ciguatera) may be useful in early treatment [101]. The recent discovery of the natural antagonist of brevetoxin known as brevenal produced by *K. brevis* may also prove to have therapeutic value in the treatment of NSP, although research to date has focused solely on the therapeutic use of brevenal for brevetoxin inhalation toxicity in laboratory animals [79].

The differential diagnosis associated with NSP includes simple food poisoning, seafood allergy, Paralytic Shellfish Poisoning (PSP), ciguatera fish poisoning, pesticide poisoning, an acute psychiatric event, or alcohol intoxication. A careful food history is critical to the correct diagnosis of this disease.

Current knowledge of NSP may be in part limited due to its tendency to be misdiagnosed and under-reported. Diagnosis may also be aided by the testing of leftovers of an implicated shellfish meal (as described below), or the collection of urine samples within a few hours of exposure. Samples of shellfish taken from the implicated shellfish harvesting area may also be used when no leftover meals exist for analysis.

## 6. Analysis of Brevetoxins

The diagnosis of NSP may be confirmed based on appropriate signs and symptoms, and a positive finding of brevetoxins in biological samples. The ability to detect, identify and quantify brevetoxins is key to gaining a better understanding of the health impacts from ingestion of contaminated molluscan shellfish. Only through the confirmation of exposure by positive chemical and biological methods (in addition to the historically important, but blunt, mouse bioassay) can definitive conclusions to be made as to a NSP case. These tools are especially important in the gathering of the information needed to determine a dose/response relationship in varying susceptible human populations.

Traditionally, there have been three distinct methodologies for the assessment of brevetoxins in environmental and biological samples [102]. These include whole animal testing known as the mouse bioassay (MBA) test. The MBA is still considered the “gold standard” for shellfish toxicity assessment, and continues to be required for the re-opening of shellfish beds in Florida and other areas after regulatory closures. It involves intra-peritoneal injections of an extracted sample of the putatively contaminated shellfish to elicit a toxic response [103]. Results are reported in mouse units (MUs) per 100 grams of shellfish. One unit is the amount of crude toxin that on the average will kill 50% of the test animals in 930 minutes [104, 105].

In the US, analysis of shellfish for brevetoxins has been driven by the regulatory framework in place designed to protect the public health. Current US guidelines state that shellfish demonstrating toxicity values of equal to or greater than 20 MUs per 100 g of shellfish (1 MU = 4.0 μg PbTx-2) are considered toxic. This is equivalent to an action level of 80 μg PbTx-2 per 100 gram of shellfish tissue (or 0.8 mg/kg or 20 MU per 100 gram of shellfish tissue). The affected harvesting areas cannot be reopened until the values fall below the required thresholds [103, 104].

The MBA has a number of drawbacks. The MBA usually takes up to 3 days to complete, making it slow in reporting and limiting the agencies’ ability to quickly respond. Shellfish beds remain closed while awaiting toxicity results. The threat of NSP due to brevetoxin-contaminated shellfish may have passed yet beds cannot be reopened until appropriate MBA results are back. This additional closure time may negatively impact the industry. In addition, the test is not specific to which toxins are responsible for the observed effects (mortality) and therefore the results are simply reporting the number of units necessary to cause mortality in a portion of the mice. The MBA also involves testing live and intact animals which requires a research facility with USDA approval, certification and inspections, making it a costly method to maintain.

More recent methods have been developed using *in vitro* methods for the detection of brevetoxins. The enzyme-linked immunosorbent assay (ELISA) for brevetoxin is an antibody based assay and is based on the activity of Goat anti brevetoxins polyclonal antibody. This antibody has a high affinity for brevetoxins which then allows for the detection and quantification of brevetoxins at very low levels. Naar and colleagues [106] reported on the successful use of the ELISA for the evaluation of seawater, shellfish samples, and mammal biological specimens (including urine and serum). Detection limits are 20 ng of toxin/gram of shellfish meat; limits in urine are 0.05 ng of toxin/ml of urine and .0.1 ng of toxin/ml of serum. The ELISA is an important tool due to its rapid assessment capabilities, low detection levels, inexpensive costs, satisfactory use by less skilled scientists, and the elimination of the need for animal bioassay studies. Its ability to detect brevetoxins at low levels will be critical to the derivation of a human dose-response curve. Detection of brevetoxins by ELISA is being used in greater frequency for research studies; however, the MBA remains the standard for regulatory assessments at this time [82, 107]. In a multi-laboratory comparison of mouse bioassay results with other methods utilizing shellfish extracts with various known concentrations of PbTx-3, the ELISA favorably compared with the mouse bioassay and could be a suitable replacement test [108].

The third traditional method for detection, identification and quantification of brevetoxins takes advantage of recent advancements in analytical chemistry. Hua [102] notes the availability of high performance liquid chromatography (HPLC) utilizing standards for the comparison of elution times. When used alone there are limitations due to co-eluding complexes. However, paired with mass spectrophotometry (MS) detectors, a definitive identification can be made. This research method is used most frequently on samples from seawater and ocean air to document and quantify the presence of red tide toxins and the relative concentrations of the various brevetoxins (PbTx-1, PbTx-2, etc.). Twiner and colleagues [109] compared the various extraction and analyses techniques used to detect and quantify brevetoxins from seawater. HPLC-MS (or LC/MS) has also been used on urine, but there are little comparable data to interpret the results.

Another method under current use includes the receptor binding assay (RBA). In this method, an unknown quantity of non-radiolabeled brevetoxin functionally competes with radiolabeled brevetoxin for the site 5 receptor of the VGSC. The biological receptor is prepared from rat brain crude membranes. Competition curves for assay calibration are generated using a range of PbTx-3 concentrations. Results provided by this assay are expressed as equivalent of PbTx-3 concentrations [109]. However, in a large multi-laboratory study comparing the mouse bioassay with other methods in shellfish extracts with known quantities of PbTx-3, the RBA preformed as well as the ELISA and was considered as a potential alternative to the MBA for the detection of brevetoxins in shellfish [108].

Radioimmunoassay (RIA) is another technique used to detect and measure brevetoxins. RIA is based on the binding of a PbTx-2 antibody that is able to recognize multiple brevetoxin congeners; however, this antibody binding does not appear to recognize all the forms of brevetoxin detected by LC/MS. Differences may be due to antibody crossreactivity of various brevetoxin congeners. Nevertheless, RIA was shown to detect a greater proportion of total brevetoxin than the RBA method in seawater [109]. RIA and ELISA are very similar methods, the RIA was developed first but with the more recent development of the ELISA this method is much less used, particularly as RIA is more difficult to perform.

In *K. brevis* red tides, the major brevetoxin produced in the seawater is PbTx-2, with lesser amounts of PbTx-1, PbTx-3 and other components produced in varying small amounts [50]. The brevetoxin profile changes with differing environmental conditions, with the stage, and the age of the red tide bloom [52, 80]. The mixed brevetoxin congener profiles in *K. brevis* blooms will produce differing results depending upon the method used for their detection and the medium sampled (e.g., seawater versus shellfish). The choice of testing method for brevetoxins is therefore dependent upon the purpose of testing (regulatory versus research) and the medium sampled. Twiner and colleagues noted that the available detection methods are not equal in their ability to measure naturally produced brevetoxins, and that most methods are hampered by the absence of specific reference standards for brevetoxin congeners. LC/MS is the only test capable of confirming the presence of a specific toxin in a specific sample, while ELISA and RBA are able to measure the overall toxins concentration in a specific sample without indication of a specific toxin. However, these other methods need to be used in conjunction with LC/MS because LC/MS is not able to infer toxicity or biological activity [109].

## 7. Epidemiology of NSP

The epidemiology of NSP is not well documented as it is a relatively rare disease with few cases reported in the published literature. Most references consist of a brief overview of the disease and the causative agent [110–112]. The relative rarity of NSP is presumed to be primarily due to the very successful regulation of commercial shellfish beds through the routine monitoring for *K. brevis* and brevetoxins in Florida and other Gulf of Mexico coastal states where *K. brevis* most often occurs. New Zealand also monitors for brevetoxins and other marine toxins as do most other potentially affected international harvesting areas. There have been few outbreaks in recent years although Florida sometimes receives a small number of sporadic case reports usually following a pronounced *K. brevis* red tide event. NSP is a reportable disease in Florida; treating physicians and laboratories are required by statute (Chapter 64D-3.002 (1) qq, Florida Administrative Code) to report cases to the Florida Department of Health; these reporting requirements allow Florida to have a somewhat better picture of the disease, currently and historically, than is available in most other states. Despite these requirements, it is generally thought that NSP is under-reported as are many foodborne and waterborne illnesses [113].

Historical reports of illness related to the consumption of shellfish harvested during or just after a red tide may include cases of NSP, but historical surveillance data are not detailed enough to confirm this. “Tainted shellfish” related illness was documented in 1880 with dead birds and fish kills also noted during this same period in Tampa [10]. It was not until the 1950s that the relationship between red tide events and NSP became better understood and researched [59, 114]. Scattered reports of cases have occurred in Florida in recent years with 2 cases in 1995, 3 in 1996, 2 in 2001, and 4 in 2005 [69, 115]. Many reports of NSP involve a single case or small case series [59–61, 116] with few large outbreaks recorded.

The largest documented outbreak of NSP occurred in New Zealand in 1992–1993 with over 180 cases reported over a period of several weeks. Although brevetoxins were implicated, more than one group of marine toxins and more than one algal species appear to have been involved [36]. Green mussel, cockles and oysters were implicated in the New Zealand clusters [33, 36–39, 85, 117].

The largest and best documented outbreak in the United States occurred in North Carolina [30]. It began in October 1987 when a *K. brevis* bloom became entrained in the Gulf Stream off eastern Florida and was transported up the eastern seaboard [30, 118]. Satellite imagery showed a strong inshore eddy of the Gulf Stream which deposited the bloom onto the North Carolina coast where it rapidly proliferated. At that time, routine monitoring for brevetoxins was not done in North Carolina and red tide was not usually seen there. Forty eight cases were identified in the ensuing weeks. North Carolina closed shellfish beds and produced public news releases and alerts as soon as the red tide was documented. However, most cases occurred prior to or just after these official actions when public health information had not yet been widely disseminated. Further investigation implicated oysters in 19 of 20 shellfish meals and clams with oysters in the other case meal. Samples of oysters from 2 implicated meals were obtained, and elevated brevetoxins were found (35 and 60 MU per 100 g of tissue). Analyses of oysters harvested from the general areas affected by the red tide were also found to be toxic (mean MU = 62, range of 48–170). Illness was observed in a few cases where less than 12 oysters were consumed, but the attack rate was 65% among those eating 12 oysters or more [30]. Gessner [119] calculated a low but not minimum toxic dose equivalent based on this outbreak as 42–72 MUs.

The most prevalent symptoms reported were: paresthesia (81%), vertigo (61%), malaise (50%), abdominal pain (48%), nausea (44%), diarrhea (33%), motor weakness (31%), and ataxia (27%) [30]. Interestingly, 17% reported temperature reversal in this outbreak, a symptom not commonly reported in Florida outbreaks and more commonly associated with ciguatera fish poisoning. Mean time to onset was 3 hours (range 15 minutes to 18 hours), and a dose response was associated with meal size. Nearly all cases had multiple symptoms, and most had more than one neurological symptom. Only 1 case in the outbreak was admitted to the hospital for severe neurological effects: bilateral carpopedal tremor, myalgias, total body paresthesia, ataxia, and vertigo. There were no cases involving respiratory distress in the North Carolina outbreak. Morris [30] reported a mean duration of illness of 17 hours (range 1–72 hours). This outbreak remains the best clinical and epidemiological description of an NSP outbreak reported in the peer reviewed literature. Routine monitoring of shellfish beds for *K. brevis* has now been established in North Carolina based in part on the success in reducing the number of cases after the bloom was identified and warnings issued, as well as the success of Florida’s monitoring programs.

Although the North Carolina outbreak was large, there were few hospitalizations and overall few serious symptoms. In contrast, Florida has documented very severe clinical presentations of NSP in recent years. In 2005, four individuals were seen in hospitals and diagnosed with NSP after a meal of oysters harvested out of an area that was closed due to elevated *K. brevis* cell numbers. Two of the cases were children (ages 6 and 9), and the youngest experienced seizures and required intubation in an intensive care unit. Other symptoms reported were involuntary muscle spasms and cramps (all cases), abdominal cramps, and paresthesia in the face and extremities (reported for 3 of 4 cases), and vomiting and headache (for 2 of 4 cases). The onset was rapid, and the children were more seriously affected than the adults [86].

Another serious incident occurred when a family of three was poisoned in June of 1996 after the consumption of self-harvested shellfish (whelks and clams) collected in Sarasota Bay (Florida). This was the first documented illness associated with whelks in Florida. The parent experienced paresthesias in the face and extremities, and vomiting. The two small children (ages 2 and 3) were hospitalized with severe neurological signs and symptoms (including seizures, convulsions, loss of consciousness, shortness of breath, and tachycardia). Vomiting and abdominal pain were also present. Both children were placed in the intensive care unit; recovery reportedly occurred within a few days although no long term follow up was performed. Brevetoxins were confirmed in the urine of the children via RIA (i.e. 42 [±2] ng/ml brevetoxin-like activity in one, and 117 [± 30] ng/ml in the second child). These are some of the few reported RIA results for humans post exposure as testing is not routinely done. Brevetoxins were detected by RIA in shellfish extracts as well [41, 68]. The father reported eating several whelks, and the children ingested unknown but presumably smaller amounts. In this particular outbreak, the poisoning is thought to have occurred after a small meal, and the symptoms and toxicity appeared to be a function of dose (with body weight being an important variable).

In the time period from June 1996 to May 1997, the mouse bioassay results for clams (*Chione cancellata* and *Mercenaria campechiensis*) collected in that same geographic area ranged from a high of 95.1 MU/100 g shellfish (the month of poisoning, June 1996) to below detection limits, but generally remained elevated. The researchers specifically noted that in some cases where the toxin levels fell below 20 MU, the mice still demonstrated classic brevetoxin signs suggesting that although the bioassay may record <20MU/100 g shellfish (and thus be considered safe), there may still be brevetoxins present [41]. Specimens of *C. cancellata* were positive for NSP up to one year past the bloom, demonstrating toxicity long past the commonly reported depuration period of 4–8 weeks noted in commercial species. As clams can be a food source for other shellfish (such as some whelks), bioaccumulation in the food chain is occurring.

Subsequent work explored the toxin retention time in the species of whelk (*Busycon contrarium*) implicated in these poisonings. One month post-incident whelks and two species of co-occurring clams were collected from this location. RIA analysis revealed that the toxin concentrations varied with the species tested (i.e. whelks were the most toxic). Both species of clams also contained brevetoxins, but at lesser concentrations. Differences in the concentration by species may be related to the differences in feeding strategies (reflecting food-chain magnification of toxins), assimilation rates, and depuration rates. The authors noted that the regulatory limit for brevetoxins in shellfish is 0.8μg/g; whelks and one species of clam failed regulatory limits, while one clam species passed. This highlights one of the risks to public health that single species testing creates [41, 68].

A more recent outbreak occurred in southwestern Florida in 2006 during a prolonged *K. brevis* red tide bloom. To date, 20 cases have been reported to Florida health officials (additional cases have been provisionally identified and are not included in this discussion). The bloom had been particularly long lasting and caused shellfish bed closures from January 2005 through January 2006; Florida red tide conditions reappeared and the closures resumed in June of 2006 through December of 2006 with patchy to heavy impacts reported all through 2006. Cases of NSP appeared from March through December of 2006 in sporadic fashion, with the majority being recorded in July of 2006 [69, 87].

The severity of symptoms in this 2006 Florida outbreak appeared to be more serious than reported in the larger 1997 outbreak in North Carolina. Data from the Florida disease registry, from the Florida Poison Information System and from telephone administered questionnaires were used to obtain clinical and demographic data for cases. Hospital records were abstracted for those cases seen in the emergency department or admitted to hospitals. All the Florida cases reported multiple symptoms (range in number of symptoms reported was 5–17) including neurological symptoms. This outbreak was categorized by a series of sporadic small clusters of cases, rather than cases appearing in a defined period. The most common symptoms cases (n=20) reported were paresthesias in the lips and mouth (90%); paresthesias in the extremities (90%); nausea (80%); muscle weakness (80%); vomiting (65%); ataxia (65%); slurred speech (55%); dizziness (50%); and respiratory discomfort (35%). Diarrhea, fatigue, pain, muscle contractions, headache, cramping, partial paralysis and severe neurological effects were reported to a lesser degree (in 20–30% of cases). Chest pain, blurred vision, sweating, respiratory distress, tachycardia and fever were not often reported (in 10% or less of cases). Seventeen of the 20 individuals sought medical treatment at local emergency departments of which 7 (41%) were admitted to the hospital. One individual with underlying medical conditions was placed on ventilatory support and experienced a reportedly full recovery after 3 days. Another individual was placed in the intensive care unit for a short period with severe neurological symptoms. In addition to the more common symptoms mentioned above, some individuals reported spasms of uncontrollable muscle contractions and psychotic-like outbursts (n= 6). Only one case in this Florida series reported temperature reversal sensations. Differences in reported symptoms between the Florida and the North Carolina outbreaks may be due, in part, to variations in the methods used to document symptoms and to the longer lag time between disease and interview for some of the later reported Florida cases. Variations in the composition of the brevetoxin mixture (potency of toxin) between these two red tide events may also be responsible for symptom differences, although this theory cannot be retrospectively tested.

Leftover clam samples were available for testing from a few of these 2006 Florida cases. Total brevetoxins were 42.9 ppm and 24 ppm (based on the brevetoxin ELISA) in one cluster, considered well above the 20 MU/100g action level. Composite analysis of clams harvested from the same general area as the implicated clams were also positive at 23.6 ppm via ELISA. Samples of clams from additional clusters of human cases also came back positive with PbTx-3 levels far in excess of the mouse bioassay guidance equivalents for this toxin at 0.8 ppm. Testing of urine from various cases yielded some positive results for sodium channel activity which aided in diagnoses and confirmation of these cases. Nevertheless, there are little data available (LC-MS) for brevetoxin and its metabolites in urine.

Risk factors identified for the Florida outbreak included being a tourist and Asian. All cases in this outbreak were linked to recreationally harvested shellfish, mostly clams. It is thought that tourists in general may be particularly unaware of the rules and regulations associated with shellfish harvesting as well as being unaware of recent red tide events in the area. It is not known why this outbreak contained a number of tourists of Asian descent except that there may be cultural practices and eating preferences that play a role (in addition to the issues of being a tourist in general). Individuals reported being unaware of the harvesting status of particular shellfish beds, and had no knowledge of where or even why shellfish beds were monitored and regulated. County health officials had issued numerous and diverse public messages and alerts once the outbreaks were detected in July. Although the number of cases of NSP did lessen, sporadic cases appeared throughout the following months. Providing effective education and outreach messages to transient populations (such as tourists and to individuals with diverse cultural and linguistic backgrounds) presents an ongoing challenge in a tourist-destination state such as Florida. Healthcare provider awareness about *K. brevis* red tide and its health risks should have been high given that Florida red tide is nearly endemic in this area, and the fact that the outbreak occurred during a prolonged period of red tide that had received notable amounts of press. Nevertheless, NSP was initially misdiagnosed in approximately 60% of the cases in 2006, highlighting the need for continuing medical education even in high risk geographic areas where NSP is known to occur. Cases of NSP were most commonly misdiagnosed as PSP, food poisoning or ciguatera.

The severity of symptoms and the large percentage of cases which sought medical treatment during this outbreak do not support the categorization of NSP as a relatively “mild” illness and its presentation in Florida suggests a moderate categorization (with more severe presentation possible in cases with other underlying medical conditions). It is possible that the composition and breakdown of the various brevetoxins among different Florida red tides has an effect on the range of symptom severity. The actual compositions of each of the brevetoxins in the particular red tides are not known so this hypothesis cannot be explored.

### 7.1 Brevetoxin-associated Respiratory Effects

Florida red tides are also associated with other diseases in humans and mammals. Because *K. brevis* is an unarmored dinoflagellate, it is relatively fragile and easily lysed by wave and wind actions. The lysed cells release toxins into the water column causing massive fish kills. These fish kills are frequently one of the first coastal warning signs of a red tide bloom. The fragility of *K. brevis,* and the subsequent release of brevetoxins facilitates the toxin to be aerosolized by coastal wind, wave and surf conditions causing a characteristic red tide respiratory reaction. Respiratory effects associated with aerosolized red tide were first reported in the literature prior to the discovery of the causal organism [120]. Throat irritation, sneezing, coughing, itchy and watery eyes, and burning of the throat and upper respiratory tract are common in beach goers during a red tide which is being blown onshore [80, 83, 121, 122].

Ongoing research is expanding the knowledge of respiratory effects of brevetoxins. Asthmatics have been shown to have a statistically significant increase in reported symptoms and a measurable (statistically significant but not clinically significant) decrease in pulmonary function after just one hour of beach exposure to aerosolized brevetoxins. These results are from long-term follow-up of a cohort of asthmatics recruited in the Sarasota, Florida area and based on measurements taken pre - and post-one hour walk on the beach during active red tide periods (exposed) and non-active periods (non-exposed). Based on this large ongoing multi-discipline study, current recommendations are that asthmatics and those individuals with other respiratory diseases (such as chronic obstructive pulmonary disease or COPD) should not spend prolonged time on the beach during a red tide; other persons can use protective masks if necessary to visit beach areas, and should seek an air-conditioned environment off the beach if symptoms develop and/or worsen. Respiratory effects of red tide are more completely discussed in recent literature and reviews [83, 121, 123–126]. Non-asthmatics also experience throat irritation, sneezing, coughing, itchy and watery eyes, and burning of the throat during a red tide.

The Florida Poison Information Center (PIC) located in Miami, in conjunction with the Florida Department of Health, maintains an Aquatic Toxins Hotline (888-232-8635) providing information on Florida red tide and other marine toxins as well as medical advice on the management of red tide-associated illnesses. During active red tides in Florida, the PIC regularly receive hundreds of calls per month related to respiratory effects of red tide among beach goers, coastal residents and workers [127].

### 7.2 Marine Animal Brevetoxin-associated Illness

In addition to the human health effects associated with exposure to brevetoxins, marine mammals, fish, seabirds and turtles may also succumb to brevetoxin poisoning during and just after a *K. brevis* red tide [128]. There has been a long record of sea animal associated deaths due to Florida red tides in the Gulf of Mexico. During a long red tide event in 1946–1948 and in 1953–1955, large mortality among bottlenose dolphins (*Tursiops truncates*) was recorded; and historically manatee deaths (*Trichechus manatus latirostris*) have also been noted to occur coincident with red tides [129–131].

In 1996, 149 manatee deaths were attributed to Florida red tide. In previous instances, manatee deaths had been primarily associated with the consumption of brevetoxin-contaminated tunicates based on the examination of manatee stomach contents and timing of death [132]. In this 1996 episode, the route of exposure appeared to be different. Healthy appearing animals died rapidly within hours to days of first experiencing the bloom. The presence of brevetoxins in nasal and lung tissue implicated an inhalation route (aerosolized brevetoxins). Although exposure to brevetoxins in manatees can also occur through toxic food ingestion and seawater ingestion, manatees must breathe at the air to sea interface and would therefore receive a large toxic dose through inhalation [132, 133].

Dolphin deaths have also been reported, most notably the significant mortality event of 107 bottlenose dolphins in 2004 [5, 134]. Necropsy showed gut contents of menhaden (*Brevoortia spp*.) laden with greatly elevated brevetoxin concentrations [5]. Thirty four endangered Florida manatees also succumbed to Florida red tide in the spring of 2002, and were included in the aforementioned necropsy study. Both menhaden and seagrass, *Thalassia testudinum*, were found to be brevetoxin-contaminated. The menhaden were the primary stomach content in these dolphins, and the fish samples were found to be most highly contaminated with brevetoxins. Subsequent experimentation demonstrated that the brevetoxins accumulate in the viscera and muscles of both omnivorous and planktivorous fish. Despite this, these fish remained apparently healthy and available as a food source. These were considered unexpected findings. Until this study, fish were considered to be unable to transfer brevetoxins up the food chain given that fish mortality frequently occurs immediately during a Florida red tide of even low concentrations of *K. brevis* cells and/or brevetoxins in the seawater [5, 47, 134]. In addition, tissue samples (lung, liver, blubber, kidney) from dolphin carcasses categorized as not recently exposed to a red tide bloom revealed detectable levels of brevetoxins. This may imply toxin retention in these dolphins and/or continued exposure through the consumption of finfish which had accumulated and retained brevetoxins [134].

Common wisdom has suggested that NSP has been restricted to the consumption of affected molluscan shellfish usually from recreationally harvested shellfish. The results of Naar and colleagues [47] raise the possibility that humans may theoretically be exposed to brevetoxins via the consumption of healthy appearing finfish during or just after a Florida red tide event; and that fish caught in areas remote from a red tide or post bloom may be contaminated at lower concentrations. Current recommendations and guidelines do not limit the consumption of healthy appearing finfish during or after a red tide. Further research is needed to determine if there are species of finfish that should be avoided during or after a Florida red tide, or if there are windows of time around a bloom that should contain harvesting restrictions of certain finfish.

## 8. NSP: Current Regulations and Public Health Prevention

A NOEL for brevetoxins in humans has not yet been established. although toxicity occurs in the nanomolar concentration range [50, 70]. The Food and Drug Administration (FDA) action level for brevetoxins in shellfish is 20 MU/100 grams of shellfish tissue (http;www.cfsan.fda.gov/~ear/nss3-42d.html). It is clear that the consumption of even a few contaminated shellfish may result in poisoning and that the severity of the disease may be dependent upon many factors (including dose, bodyweight, underlying medical conditions, and age of the victim as well as possibly the toxin mixture of the particular bloom).

In 1925, the Surgeon General of the U.S. Public Health Service called for a conference on shellfish sanitation in response to the human health issues and to requests from various state and local agencies. Based on the recommendations from this conference, the National Shellfish Sanitation Program (NSSP) was formed in 1925. The Manual of Operations continues to provide guidance to states and agencies in managing shellfish resources. In 1982, the Interstate Shellfish Sanitation Conference (ISSC) was organized, and under its auspices uniform guidelines have been created. Currently, the ISSC has a formal agreement with the FDA that requires its members to have laws and regulation in place that ensure the safety of shellfish harvesting, growing and processing (http://www.cfsan.fda.gov~ear/nsspman.html; http://www.issc.org).

It is under these guidelines that commercial shellfish beds are closely monitored in Florida and other states by state regulatory agencies. In Florida, shellfish beds are closed when *K. brevis* cell counts reach or exceed 5,000 cells per liter of seawater given that background levels are considered to be 1,000 cells per liter of seawater or less. Shellfish beds are not reopened until the cell counts fall below 5,000 cells per liter of seawater and until mouse bioassay units fall below 20 MU per 100 g of shellfish tissue. These values are outlined in Florida’s Biotoxin Control Plan, and are highlighted on the Florida Department of Agriculture and Consumer Services aquaculture website (http://www.floridaaquaculture.com) [135].

Shellfish beds and open water areas that are not monitored and regulated by state authorities are classified as prohibited to shellfish harvesters as a means of protecting the public. Approved zones are remarkably safe, and there has not been a documented case of NSP (in Florida records) attributed to commercially regulated shellfish. In Florida, approved harvesting zones are monitored by sampling the predominant commercial shellfish in that zone (primarily clams or oysters). Most developed countries with coastal areas maintain some sort of monitoring and health alert system to mitigate the effects of harmful algal blooms [70, 104, 136, 137]. In Florida, with the best documented NSP reporting system, all reported cases of NSP have occurred when recreationally harvested shellfish have been gathered and consumed without determination of shellfish bed harvesting status or without knowledge of red tide events in the area [69, 87]. Because testing is restricted to oysters, clams or mussels, recreational harvesters may be at risk for NSP by harvesting non-commercial and thus untested species such as whelks, as well as by harvesting in beds that are always prohibited or currently closed due to elevated brevetoxin values.

Prevention of NSP cases is best managed through primary prevention efforts, particularly preventing consumption of contaminated shellfish. Reduction or elimination of risk is accomplished when recreational (as well as commercial) harvesters of shellfish check with authorities on the harvesting status of the beds, only harvest from officially open and regulated beds, and are aware of the history of *K. brevis* blooms in the area. Individuals should not buy or accept shellfish from unlicensed vendors or friends when they are not certain of the area of harvesting or on-going monitoring results. This primary form of prevention has successfully protected consumers of commercial shellfish. Efforts need to be made to more clearly highlight shellfish bed harvesting regulations as well as open and closed status in a manner that is easily and readily accessible and understandable to tourists (including foreign tourists). This knowledge would help to decrease the number of NSP cases as nearly all cases in recent years have been associated with recreational harvesting. Changes in disease surveillance practices, particularly during a red tide bloom event, may provide timely alerts to an outbreak and would allow better estimates of disease incidence. Use of nontraditional sources of disease data such as available from Poison Information Centers would provide a beneficial partnership as these calls are sometimes an early alert concerning a cluster or outbreak of disease (NSP) [137, 138].

Research continues on numerous related topics including: the identification and geographic distribution of brevetoxin-producing species; variability of species that produces brevetoxins; variability of brevetoxin depuration times among molluscan species; accumulation and depuration of brevetoxins in finfish and their potential role in human and mammalian toxicity; acute and chronic human health effects; and the toxicology of brevetoxins and brevetoxin metabolites (including antagonists such as brevenal). As new information is gained from this research, prevention strategies may be updated to accommodate these findings.

## 9. Recommendations

The following activities would improve the protection of public health and reduce the incidence of NSP:

- Environmental Research:
  - Identify additional species of HABs which produce brevetoxins.
  - Determine additional species of molluscs that accumulate brevetoxins (and that are a potential food source) and their depuration rates.
  - Identify species of finfish that accumulate brevetoxins (and are a potential food source) and the distribution of the toxin within fish organs and tissues.
  - Review regional monitoring and analyses practices for mollluscs and finfish to determine the adequacy of current guidelines and protocols for protection of public health.
  - Evaluation of ELISA brevetoxin method as potential replacement of the mouse bioassay.
- Surveillance:
  - Increase and improve public health disease surveillance of NSP and other marine toxins for better estimates of disease incidence.
  - Provide timely alerts to public health epidemiologists during an outbreak to improve detection of NSP-associated illness.
  - Increase number of states that designate illnesses related to marine toxins (NSP, Ciguatera, etc.) as reportable diseases to improve surveillance; obtain better estimates of national incidence of HAB-related illness and to develop appropriate prevention strategies.
- Outreach and Education Information
  - Improve dissemination of information on shellfish harvesting beds and regulations including harvesting openings and closings.
  - Develop easily available user friendly web sites and related materials for the general public including transient populations such as tourists and part time residents.
  - Produce targeted educational campaign on signs, symptoms and diagnosis of NSP for first responders, emergency department physicians, and other healthcare providers, particularly in high-risk areas.
  - Develop targeted education on the signs, symptoms and diagnosis of NSP for first responders, emergency department physicians, and other healthcare providers, particularly for high-risk areas.
  - Ensure education and outreach activities have components tailored to reach transient populations including non-English speaking visitors in high-risk areas.
- Human Toxicology and Epidemiology
  - Improve understanding of brevetoxins (and metabolites) toxicity, with including minimal doses for adverse effects, dose response curves, with emphasis on human exposures.
  - Evaluate efficacy of mannitol or brevenal for treatment of NSP.
  - Follow confirmed NSP cases over a longer period of time to determine whether there are long term adverse effects or chronic sequela to exposures.
